# SPICT tool among intubated elderly patients at emergency department

**DOI:** 10.1016/j.heliyon.2024.e39905

**Published:** 2024-10-30

**Authors:** Thanat Tangpaisarn, Ponpich Prajammuang, Sukanya Khemtong, Pariwat Phungoen, Phraewa Thatphet

**Affiliations:** Department of Emergency Medicine, Faculty of Medicine, Khon Kaen University, Thailand

**Keywords:** Intubation, Elderly, Palliative care, Emergency department, Mortality, SPICT

## Abstract

**Background:**

The global rise in the aging population necessitates a proactive approach to palliative care, with a substantial gap between the demand for and access to such care. The Supportive and Palliative Care Indicators Tool (SPICT) has emerged as a valuable instrument for identifying patients at risk of deterioration and death, enhancing the timely initiation of palliative care. This study aimed to investigate the characteristics and outcomes of patients aged 60 and above who got intubated in the emergency department (ED), with and without fulfilling SPICT criteria.

**Methods:**

This retrospective single-center study was conducted at a tertiary care teaching hospital in Thailand. The research involved 408 adults aged 60 and older who underwent ED intubation and subsequent admission. Baseline characteristics and follow-up data were collected, encompassing vital signs, comorbidities, and SPICT criteria. Mortality rates between SPICT criteria-fulfilled and non-fulfilled groups were compared.

**Results:**

Out of 408 analyzed patients, SPICT criteria were met by 39.7 % of patients, exhibiting distinctive features such as a higher respiratory rate and lower diastolic blood pressure. Hypertension and diabetes mellitus were most prevalent comorbidities. Respiratory and neurological disorders were the leading final diagnoses. The overall in-hospital mortality rate was 28 %, significantly higher in the SPICT criteria-fulfilled group compared to the non-fulfilled group (34.0 % vs 23.6 %, P = 0.022). SPICT criteria demonstrated moderate sensitivity (48.7 %) and specificity (63.7 %) for predicting mortality.

**Conclusion:**

SPICT criteria identified elderly patients at an elevated mortality risk following intubation in the emergency setting. The early implementation of SPICT as a screening tool for identifying palliative care candidates is advocated for more effective advance care planning.

## Highlights

• Elderly intubated patients meeting SPICT criteria have higher in-hospital mortality.

## 1 Introduction

Every country is experiencing growth in both the size and proportion of elderly in the population [1]. The global demand for palliative care is expected to rise in the coming years, owing to an aging population [2]. Worldwide, only about 14 % of people who need palliative care currently receive it indicating a significant gap between the number of those requiring this service and those with access to it [2]. Early palliative care reduces hospital admissions and healthcare utilization, improving patient outcomes and resource utilization [2].

Worldwide, over 56.8 million people are estimated to require palliative care every year including 31.1 million prior to and 25.7 million near the end of life. The majority (67.1 %) are adults over 50 years old [3]. Assessment criteria have been developed to expedite access, reducing unnecessary hospitalizations [4]. Supportive and Palliative Care Indicators Tool (SPICT) was developed in the United Kingdom to help healthcare professionals identify patients at risk of deterioration and death and support them in timely initiating palliative care [5]. Since its development in the UK, the SPICT tool has been increasingly adopted in various healthcare settings globally, including in Europe, Australia, and Asia, demonstrating its utility in identifying patients for palliative care across diverse populations [5,6]. Its broad applicability underscores the value of SPICT in guiding early palliative care interventions in different cultural and clinical environments. The SPICT tool uniquely incorporates functional decline as a key criterion, which sets it apart from other commonly used hospital mortality predictors, such as the Charlson Comorbidity Index and APACHE II, which predominantly assess comorbidities and physiological parameters [5,7]. Functional decline, often seen in elderly patients with chronic illnesses, can provide earlier signals of the need for palliative care, supporting timely interventions. The SPICT is composed of general indicators of health deterioration as well as disease severity indicators [7,8].

Several studies among patients with cancer found that the patients who enter palliative care or hospice were less likely to visit the ED [[9], [10], [11], [12], [13]]. Early entry into palliative care, for patients meeting the criteria, could reduce ED overcrowding by potentially preventing unnecessary acute admissions.

Older adult patients tend to have higher emergency department (ED) visit rates, as well as admissions rates compared to younger age groups [13]. Unfortunately, after emergency intubation, 33 % percent of older adults die during hospitalization [14]. From previous studies, the factors associated with in-hospital mortality were stroke, chronic kidney failure, higher scores on the Charlson Comorbidity Index, APACHE II, and SOFA are associated with increased in-hospital mortality, as they reflect more severe illness and comorbid conditions [15,16].

This study aimed to identify the characteristics and outcomes of endotracheal tube intubated patients aged above 60 with and without fulfilling palliative care criteria at an emergency department.

## 2 Methods

### 2.1 Study design and setting

The study was a retrospective single-center study, utilizing data derived from visits to the emergency department (ED) at Srinagarind Hospital, Khon Kaen, Thailand, a distinguished tertiary care teaching center. Extensive extraction and manual review of hospital databases were conducted to cover the period from August 1, 2020, to December 31, 2022. To ensure consistent data abstraction and reduce investigator bias, two independent reviewers (PP and PT) extracted data from patient records. In cases of discrepancies, the reviewers reached a consensus through discussion. This approach enhances the reliability of retrospective data extraction by minimizing subjectivity. Ethical approval was obtained from the Center for Ethics in Human Research, Khon Kaen University (HE661125).

### 2.2 Study population

The study targeted adults aged 60 years and older who sought treatment at the ED, underwent intubation during their ED visit, and were subsequently admitted to the inpatient department at Srinagarind Hospital. Exclusion criteria encompassed traumatic cases, patients transferred to other medical facilities, and those experiencing cardiac arrest at the ED.

### 2.3 Variables

Baseline characteristics were collected during the initial ED presentation, including, age, gender, specific comorbidities (diabetes mellitus, hypertension, cerebrovascular disease, heart disease, cancer, dementia, neurological disease, liver disease, respiratory disease, and kidney disease), vital signs, and blood gas analysis. Follow-up data encompassed final diagnosis, length of hospital stay, duration of intubation, and discharge status. The categorization of patients based on the Supportive and Palliative Care Indicators Tool (SPICT) criteria was employed, where a positive SPICT assessment indicated the presence of at least one general or disease-specific indicator. (Supplementary Material) The primary outcome measure was the hospital mortality rate.

### 2.4 Sample size calculation and sampling method

The sample size was calculated using the formula for estimating an infinite proportion, considering a mortality rate of 33 % [14] and a margin of error of 5 %, yielding a minimum sample size of 340 patients. A systematic sampling approach was employed, wherein patients were listed based on their visit times, and the first patient was randomly selected, followed by every other patient in sequence.

### 2.5 Statistical analysis

Baseline characteristics and clinical data were presented as the median and interquartile range (IQR) for continuous variables and as counts and percentages for categorical variables. Continuous variable comparisons utilized the Mann–Whitney *U* test, while categorical variables were assessed using Pearson's chi-square test or Fisher's exact test. All statistical analyses were conducted using R Statistical Software version 4.2.3 (www.R-project.org, R Foundation for Statistical Computing).

## 3 Results

**Fig. 1:**
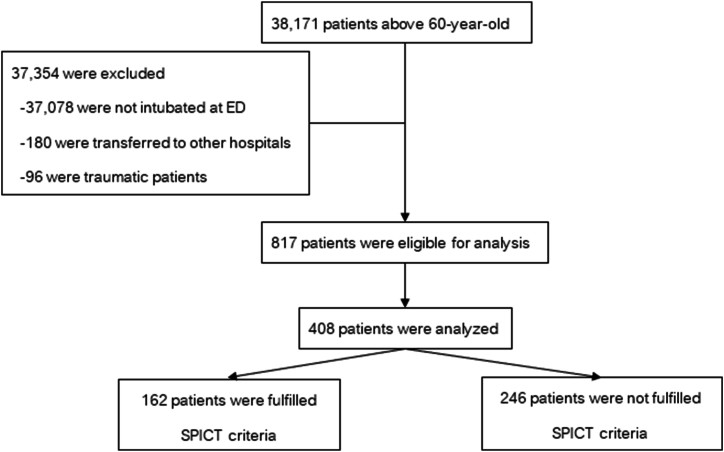
Study flow.

During the study period, a total of 38,171 patients aged 60 years and above sought medical attention at the ED. Of these, 817 non-traumatic patients underwent intubation and were subsequently admitted to the hospital. A total of 408 patients were included in the analysis (Fig. 1).

Table 1 presents the baseline characteristics of the 408 analyzed patients. The study cohort, with a median age of 74 years, comprised 59 % males, and 39.7 % met the SPICT criteria. Patients meeting SPICT criteria exhibited distinct features, including a higher respiratory rate, lower diastolic blood pressure, and lower oxygen saturation. Hypertension and diabetes mellitus emerged as the two most prevalent comorbid conditions across both groups.

**Table 1:** Patients baseline characteristics.

Characteristics	Overall, n = 408	Non-fulfilled SPICT, n = 246	Fulfilled SPICT, n = 162	p-value
Age, year, median (IQR)	74 (67–81)	73 (66–81)	76 (69–82)	0.004
Age-group				0.042
60–69, n (%)	132 (32.4 %)	89 (36.2 %)	43 (26.5 %)	
70–79, n (%)	141 (34.6 %)	85 (34.6 %)	56 (34.6 %)	
80–89, n (%)	118 (28.9 %)	66 (26.8 %)	52 (32.1 %)	
90–99, n (%)	17 (4.2 %)	6 (2.4 %)	11 (6.8 %)	
Male, n (%)	241 (59.1 %)	155 (63.0 %)	76 (53.1 %)	0.046
BT, Celsius, median (IQR)	36.9 (36.5–37.7)	36.8 (36.5–37.6)	37.0 (36.5–37.9)	0.324
HR (bpm), median (IQR)	95 (81–113)	94 (79–114)	98 (84–113)	0.148
RR (/min), median (IQR)	28 (22–32)	28 (22–32)	30 (24–36)	0.007
SBP (mmHg), median (IQR)	141 (120–165)	145 (120–166)	140 (119–160)	0.184
DBP (mmHg), median (IQR)	79 (81–113)	80 (66–96)	74 (64–90)	0.037
Hypotension, n (%)	37 (9.1 %)	16 (6.5 %)	21 (13.0 %)	0.026
O2saturation, median (IQR)	95 (87–98)	96 (88–99)	92 (86–98)	0.036
Comorbidity				
None, n (%)	37 (9.1 %)	34 (13.8 %)	3 (1.9 %)	<0.001
Cancer, n (%)	56 (13.7 %)	28 (11.4 %)	28 (17.3 %)	0.122
Cerebrovascular disease, n (%)	91 (22.3 %)	35 (14.2 %)	56 (34.6 %)	<0.001
Dementia, n (%)	32 (7.8 %)	10 (4.1 %)	22 (13.6 %)	<0.001
Diabetes mellitus, n (%)	168 (41.2 %)	89 (36.2 %)	79 (48.8 %)	0.011
Heart disease, n (%)	127 (31.1 %)	58 (23.6 %)	69 (42.6 %)	<0.001
Hypertension, n (%)	245 (60.0 %)	133 (54.1 %)	112 (69.1 %)	0.002
Kidney disease, n (%)	92 (22.5 %)	48 (19.5 %)	44 (27.2 %)	0.091
Liver disease, n (%)	37 (9.1 %)	21 (8.5 %)	16 (9.9 %)	0.776
Neurological disease, n (%)	28 (6.9 %)	10 (4.1 %)	18 (11.1 %)	0.006
Respiratory disease, n (%)	62 (15.2 %)	34 (13.8 %)	28 (17.3 %)	0.417

### 3.1 Patients’ outcome

Table 2 delineates the most prevalent final diagnoses in the SPICT criteria-fulfilled group, highlighting respiratory disorders (36 %) and neurological disorders (24 %). Conversely, in the non-fulfilled SPICT criteria group, neurological disorders (28 %) and respiratory disorders (27 %) predominated. No significant differences were observed in blood gas analysis, length of hospital stays, length of intubation, or tracheostomy rates between the two groups.

**Table 2:** Patients diagnosis and outcome.

Outcome	Overall, n = 408	Non-fulfilled SPICT, n = 246	Fulfilled SPICT, n = 162	p-value
Final diagnosis				
Cardiovascular, n (%)	83 (20.3 %)	44 (17.9 %)	39 (24.1 %)	0.164
Gastrointestinal, n (%)	44 (10.8 %)	30 (12.2 %)	14 (8.6 %)	0.333
Infection, n (%)	57 (14.0 %)	33 (13.4 %)	24 (14.8 %)	0.800
Metabolic, n (%)	4 (1.0 %)	2 (0.8 %)	2 (1.2 %)	0.651
Neurology, n (%)	93 (22.8 %)	69 (28.0 %)	24 (14.8 %)	0.002
Respiratory, n (%)	125 (30.6 %)	67 (27.2 %)	58 (35.8 %)	0.066
Other, n (%)	2 (0.5 %)	1 (0.4 %)	1 (0.6 %)	1.000
**Blood gas analysis**				
pH, median (IQR)	7.39 (7.31–7.45)	7.39 (7.31–7.44)	7.40 (7.31–7.47)	0.182
pO2, median (IQR)	88 (59–146)	85 (60–145)	92 (59–152)	0.467
pCO2, median (IQR)	32 (59–146)	31 (25, 37)	32 (27, 38)	0.210
HCO3, median (IQR)	20 (16–23)	19 (15–23)	20 (16–23)	0.122
Length of stay, day, median (IQR)	11 (7–20)	11 (6–19)	12 (8–21)	0.156
Length of intubation, days, median (IQR)	4 (3–8)	4 (3–7)	3 (3–8)	0.261
Tracheostomy, n (%)	31 (7.6 %)	20 (8.1 %)	11 (6.8 %)	0.757
Hospital mortality, n (%)	113 (27.7 %)	58 (23.6 %)	55 (34.0 %)	0.022

A total of 113 patients died during their hospital admission, yielding an overall in-hospital mortality rate of 28 %. Stratifying by SPICT criteria fulfillment, the mortality rates were 34.0 % in the SPICT criteria-fulfilled group and 23.6 % in the non-fulfilled group, representing a statistically significant difference (P = 0.022). The diagnostic performance of SPICT criteria in predicting hospital mortality is detailed in Table 3, with a sensitivity of 48.7 % and specificity of 63.7 %.

**Table 3:** Diagnostic performance of SPICT to predict hospital mortality.

Metrics	Performance (95 % CI)
Positive Predictive Value	34.0 % (27.1 %–41.5 %)
Negative Predictive	76.4 % (70.7 %–81.3 %)
Sensitivity	48.7 % (39.7 %–57. 8 %)
Specificity	63.7 % (58.1 %–69.0 %)

## 4 Discussion

Our study, a retrospective single-center investigation conducted at Srinagarind Hospital, Khon Kaen, Thailand, aimed to examine the clinical outcomes of patients aged 60 years and above who underwent intubation in the ED and subsequent admission to the hospital.

The overall mortality rate observed in our study was 28 %, aligning closely with the findings of Ouchi et al., who reported a mortality rate of 29 % in patients aged 65–74 years undergoing endotracheal intubation [14]. However, our study's mortality rate was notably lower when compared to the study by Steenebrugen et al., which reported a mortality rate of 72 %. This discrepancy may be attributed to the inclusion of patients aged over 75 years, trauma cases, and cardiac arrest patients in their study [16].

In the group meeting SPICT criteria, patients demonstrated a higher prevalence of pre-existing comorbidities, likely due to their prior engagement with the healthcare system. Significant differences were noted in the prevalence of specific comorbidities, including a history of cerebrovascular accidents, dementia, diabetes, heart disease, hypertension, and other neurological conditions. This heightened prevalence aligns with the findings of Ruiz et al. and Ouchi et al., who identified that a high Charlson Comorbidity Index (CCI) contributes to in-hospital mortality [17,15]. Identifying patients who may benefit from palliative interventions has the potential to reduce ED visits, as demonstrated in studies involving cancer patients [[9], [10], [11], [12], [13]]. Palliative care is most beneficial when initiated early in the course of a serious illness, rather than being reserved for the terminal phase. Early initiation of PC has been shown to improve quality of life, reduce hospitalizations, and enhance patient outcomes [3,18]. This approach allows for better management of symptoms and care planning, particularly in elderly patients with multiple comorbidities. Such targeted identification could contribute to alleviating ED overcrowding.

The predominant diagnoses in our patient population were respiratory and neurological conditions, consistent with the research by Steenebrugen et al. and Ouchi et al., highlighting respiratory failure and cerebrovascular accidents as primary contributors to diseases in elderly patients undergoing endotracheal intubation [14,15]. These conditions correspond to the leading causes of death in individuals aged 55 to 74 in Thailand, primarily cerebrovascular diseases and lower respiratory tract infections [16].

Our findings indicate that elderly patients meeting SPICT criteria following endotracheal intubation in the emergency department experienced a higher mortality rate compared to those not meeting the criteria, with a sensitivity of 48.7 % and specificity of 63.7 %. In contrast, a study by Mudge et al. utilized SPICT to predict 12-month mortality in inpatients, yielding a sensitivity of 78 % and specificity of 72 % [18]. The differences in performance metrics may be attributed to variations in patient age and disease severity. These results underscore the potential value of early SPICT utilization as a screening tool for identifying palliative care candidates, facilitating more comprehensive advance care planning.

Effective communication between healthcare professionals, patients, and caregivers is essential in the early identification of palliative care needs using the SPICT tool. Studies have shown that involving caregivers in discussions about prognosis and care planning can lead to more timely and appropriate palliative interventions, improving both patient outcomes and caregiver satisfaction [19]. This underscores the role of SPICT in facilitating these critical conversations in the ED setting.

Although our study provides valuable insights, the retrospective nature of our research restricts our ability to establish causality, and the single-center design may limit generalizability. Future research incorporating detailed diagnoses, and a multi-center approach could further refine risk prediction models and enhance care pathways for this vulnerable population.

## 5 Conclusion

This study emphasizes the practicality of SPICT criteria in identifying elderly patients at an elevated risk of mortality following intubation in the emergency setting. The early implementation of SPICT as a screening tool for identifying palliative care candidates is advocated for more effective advance care planning.

## Presentation

This study was presented as an abstract poster presentation at on 20–June, 2024 at 23rd International Conference on Emergency Medicine.

## CRediT authorship contribution statement

**Thanat Tangpaisarn:** Writing – review & editing, Writing – original draft, Project administration, Methodology, Formal analysis. **Ponpich Prajammuang:** Writing – original draft, Investigation, Data curation, Conceptualization. **Sukanya Khemtong:** Writing – review & editing, Formal analysis. **Pariwat Phungoen:** Writing – review & editing, Project administration, Formal analysis. **Phraewa Thatphet:** Writing – review & editing, Writing – original draft, Validation, Supervision, Data curation, Conceptualization.

## Trial registration

This study was approved by the Khon Kaen University Ethics Committee in Human Research (HE661125)

## Declaration of generative AI and AI-assisted technologies in the writing process

During the preparation of this work, the authors used ChatGPT, a language model developed by OpenAI, in order to improve the language of the manuscript. After using this tool, the author(s) reviewed and edited the content as needed and take(s) full responsibility for the content of the publication.

## Declaration of competing interest

The authors declare that they have no known competing financial interests or personal relationships that could have appeared to influence the work reported in this paper.

## References

[1] Ageing and health. https://www.who.int/news-room/fact-sheets/detail/ageing-and-health.

[2] Palliative care. https://www.who.int/news-room/fact-sheets/detail/palliative-care.

[3] Connor S. (2020).

[4] Assessing the development of palliative care worldwide: a set of actionable indicators. https://www.who.int/publications-detail-redirect/9789240033351.

[5] Highet G., Crawford D., Murray S.A., Boyd K. (2014). Development and evaluation of the supportive and palliative care indicators tool (SPICT): a mixed-methods study. BMJ Support. Palliat. Care.

[6] Mudge A.M., Douglas C., Sansome X. (2018). Risk of 12-month mortality among hospital inpatients using the surprise question and SPICT criteria: a prospective study. BMJ Support. Palliat. Care.

[7] Walsh R.I., Mitchell G., Francis L., van Driel M.L. (2015). What diagnostic tools exist for the early identification of palliative care patients in general practice? A systematic review. J. Palliat. Care.

[8] About SPICT (2014). SPICT. October.

[9] Gómez-Batiste X., Tuca A., Corrales E. (2006). Resource consumption and costs of palliative care services in Spain: a multicenter prospective study. J. Pain Symptom Manag..

[10] Alonso-Babarro A., Astray-Mochales J., Domínguez-Berjón F. (2013). The association between in-patient death, utilization of hospital resources and availability of palliative home care for cancer patients. Palliat. Med..

[11] Bergman J., Saigal C.S., Lorenz K.A. (2011). Hospice use and high-intensity care in men dying of prostate cancer. Arch. Intern. Med..

[12] Carlson M.D.A., Herrin J., Du Q. (2010). Impact of hospice disenrollment on health care use and medicare expenditures for patients with cancer. J. Clin. Oncol..

[13] Saito A.M., Landrum M.B., Neville B.A., Ayanian J.Z., Weeks J.C., Earle C.C. (2011). Hospice care and survival among elderly patients with lung cancer. J. Palliat. Med..

[14] Lumjeaksuwan M., Patcharasopit S., Seksanpanit C., Sritharo N., Aeampuck A., Wittayachamnankul B. (2021). The trend of emergency department visits among the elderly in Thailand. WHO South East Asia J Public Health.

[15] Ruiz V.R., Grande-Ratti M.F., Martínez B., Midley A., Sylvestre V., Mayer G.F. (2021). In-hospital mortality associated factors in elderly patients with invasive mechanical ventilation in the emergency department. Enfermería Intensiva.

[16] Steenebruggen F., Higuet A., Hubloue I., Beyer I. (2018). Characteristics and outcome of Emergency Department intubation in geriatric patients. Eur Geriatr Med.

[17] Ouchi K., Jambaulikar G.D., Hohmann S. (2018). Prognosis after emergency department intubation to inform shared decision-making. J. Am. Geriatr. Soc..

[18] Temel J.S., Greer J.A., Muzikansky A. (2010). Early palliative care for patients with metastatic non–small-cell lung cancer. N. Engl. J. Med..

[19] Higginson I.J., Evans C.J. (2010). What is the evidence that palliative care teams improve outcomes for cancer patients and their families?. Cancer J..

