# Supplementary material for: SPICT tool among intubated elderly patients at emergency department
Source: Heliyon. 2024 Oct 30;10(21):e39905. doi: 10.1016/j.heliyon.2024.e39905 (PMC11566668; doi:10.1016/j.heliyon.2024.e39905)
Supplement: Multimedia component 1 [file mmc1.docx]

**Supplementary Material**

**Figure S1: Supportive and Palliative Care Indicators Tool (SPICT™) 2022**^1^

| **Look for any general indicators of poor or deteriorating health.** |
| --- |
| - Unplanned hospital admission(s). |
| - Performance status is poor or deteriorating, with limited reversibility. (Person stays in bed or in a chair for more than half the day.) |
| - Depends on others for care due to increasing physical and/or mental health problems. The person's carer needs more help and support. |
| - Progressive weight loss; remains underweight; low muscle mass. |
| - Persistent symptoms despite optimal treatment of underlying condition(s). |
| - The person (or family) asks for palliative care; chooses to reduce, stop or not have treatment; or wishes to focus on quality of life. |
| **Look for clinical indicators of one or multiple life-limiting conditions.** |
| **Cancer** |
| - Too frail for cancer treatment or treatment is for symptom control. |
| - Functional ability is deteriorating due to progressive cancer. |
| **Dementia/ frailty** |
| - Unable to dress, walk or eat without help. |
| - Eating and drinking less; difficulty with swallowing. |
| - Urinary and faecal incontinence. |
| - Not able to communicate by speaking; little social interaction. |
| - Frequent falls; fractured femur. |
| - Recurrent febrile episodes or infections; aspiration pneumonia. |
| **Neurological disease** |
| - Progressive deterioration in physical and/or cognitive function despite optimal therapy. |
| - Speech problems with increasing difficulty communicating and/or progressive difficulty with swallowing. |
| - Recurrent aspiration pneumonia; breathless or respiratory failure. |
| - Persistent paralysis after stroke with significant loss of function and ongoing disability. |
| **Heart / vascular disease** |
| - Heart failure or extensive, untreatable coronary artery disease; with breathlessness or chest pain at rest or on minimal effort. |
| - Severe, inoperable peripheral vascular disease. |
| **Respiratory disease** |
| - Severe, chronic lung disease; with breathlessness at rest or on minimal effort between exacerbations. |
| - Persistent hypoxia needing long term oxygen therapy.​ |
| - Has needed ventilation for respiratory failure or ventilation is contraindicated.​ |
| **Kidney disease** |
| - Stage 4 or 5 chronic kidney disease (eGFR < 30ml/min) with deteriorating health. |
| - Kidney failure complicating other life limiting conditions or treatments. |
| - Stopping or not starting dialysis. |
| **Liver disease** |
| - Cirrhosis with one or more complications in the past year: |
| - diuretic resistant ascites |
| - hepatic encephalopathy |
| - hepatorenal syndrome |
| - bacterial peritonitis |
| - recurrent variceal bleeds |
| - Liver transplant is not possible. |
| **Other conditions** |
| - Deteriorating with other conditions, multiple conditions and/or complications that are not reversible; best available treatment has a poor outcome. |

**Reference**

1. The SPICT^TM^. SPICT. Published March 19, 2021. Accessed March 3, 2024. https://www.spict.org.uk/the-spict/
